# What do we know about lithium associated hypercalcemia?

**DOI:** 10.1192/j.eurpsy.2023.1207

**Published:** 2023-07-19

**Authors:** N. Laherrán Cantera, R. Palacios - Garrán, A. Sanchez-Guerra Alonso, C. Gutiérrez Santaló, L. I. Muñoz-Manchado

**Affiliations:** 1 Jerez University Hospital, Jerez de la Frontera; 2 Primary Care Service General Hospital of Ávila, Ávila, Spain

## Abstract

**Introduction:**

Lithium associated with hypercalcemia may mimic a psychiatric condition and be confused for a relapse of bipolar disorder. The etiology seems to be due to a reduced sensitivity of the parathyroid cells to calcium, altering the parathyroid hormone (PTH) response. Lithium as an essential monovalent cation has some structural similarity to calcium (Ca) and can interact with protein receptors. This leads to changes in the inhibitory configuration of PTH and increased serum calcium concentrations, rising the threshold necessary to suppress hormone secretion.

Lithium-induced hyperparathyroidism (HIL) is the main cause of hypercalcemia in these patients.

**Objectives:**

Based on a clinical case of lithium-associated hypercalcemia in a patient with bipolar disorder, review the existing literature and state the needs for periodic monitoring protocols.

**Methods:**

Case report and bibliographical review.

**Results:**

A 38-year-old woman, diagnosed with bipolar affective disorder at the age of 18, has been treated with lithium during which she developed secondary tubulointerstitial nephropathy as an adverse effect. Recently, she requested medical evaluation for constitutional syndrome associated with deterioration of general condition with loss of strength and difficulty in walking. Analytically, mild hypercalcemia was detected, and the study was extended to include Ca and PTH.

Chronic lithium therapy often develops mild hypercalcemia (approximately 10 to 20 percent of patients taking lithium), most likely due to increased secretion of PTH. Lithium can also unmask previously unrecognized mild hyperparathyroidism in patients with adenomas within a few years of starting therapy or induce parathyroid hyperplasia with a chronic use.

The hypercalcemia usually, but not always, subsides when the lithium is stopped. Normalization of serum calcium is more likely to occur one to four weeks post-lithium withdrawal in patients with a relatively short duration of lithium use. It is less likely in patients receiving lithium for more than 10 years.

Regarding the case to be presented, a review of the literature is carried out and the need to propose periodic calcium monitoring protocols is exposed.

**Conclusions:**

Recommendations include determination of serum calcium every 6 months, urinary calcium and creatinine every 12 months, and bone mineral density monitoring every 1 to 3 years. Regular analytical monitoring including total calcium, PTH and vitamin D, would identify patients with a tendency to hypercalcemia so that appropriate measures could be taken. So as chronic treatment with lithium can develop mild hypercalcemia, I consider it necessary to develop periodic monitoring protocols for this adverse effect.

**Disclosure of Interest:**

None Declared

